# Unraveling the hydrogeochemical evolution and pollution sources of shallow aquifer using multivariate statistical analysis and hydrogeochemical techniques: a case study of the Quaternary aquifer in Beni Suef area, Egypt

**DOI:** 10.1007/s10661-023-11206-9

**Published:** 2023-05-15

**Authors:** Hend S. Abu Salem, Mohammed Albadr, Mohamed M. El Kammar, Mohamed M. Yehia, Ahmed M. El-Kammar

**Affiliations:** 1 Geology Department, Faculty of Science, Cairo University, Giza, Egypt; 2 El-Minya Company for Drinking Water and Wastewater, El Minya, Egypt; 3 Central Laboratory for Environmental Quality Monitoring, National Water Research Centre, Al Qanatir Al Khayriyyah, Egypt

**Keywords:** Quaternary aquifer, Hydrogeochemistry, Multivariate statistical analysis, Pollution sources, Beni Suef, Egypt

## Abstract

This study integrates multivariate statistical analysis and hydrogeochemical modeling to investigate the processes controlling the groundwater composition of a shallow aquifer where increased pumping rates and anthropogenic impacts were prevalent. Eighteen groundwater samples were collected and analyzed for major elements and selected heavy metals. The data were classified on the basis of multivariate statistical analysis into three clusters: C1 (Na-Cl facies), C2 (Ca-SO_4_ facies), and C3 (Ca-HCO_3_ facies). The application of factor analysis gave four factors affecting the groundwater chemistry, namely the salinization factor, anthropogenic/secondary enrichment factor, the secondary and the micro-nutrient fertilizers, and the aluminum fertilizer factor. The hydrogeochemical study of the groundwater revealed that the processes controlling the groundwater chemistry in the study area are mainly affected by the groundwater occurrence either to the east or to the west of Bahr Youssef Canal. Generally, the dominant hydrogeochemical processes affecting the groundwater are silicate weathering, ion exchange, irrigation return flow, gypsum applications in soil, and evaporation. The groundwater quality evaluation shows that water quality varies from fair to excellent for drinking purposes, where the best water is located in the northern and central parts of the study area. The suitability of groundwater for irrigation was evaluated using several indices indicating that groundwater is suitable for irrigation in the northwest and western parts of the study area. As some groundwater samples lie in high salinity classes on the US Salinity diagram, it is recommended to use this water for plants with good salt tolerance under good drainage conditions. The integration between the statistical and geochemical tools helps reveal the dominant processes through data reduction and classification.

**Supplementary Information:**

The online version contains supplementary material available at 10.1007/s10661-023-11206-9.

## Introduction

Groundwater represents part of the total water resources used for domestic, agricultural, and industrial activities especially at increasing water scarcity and surface water quality deterioration in arid and semi-arid areas (Keesari et al., 2014; Tsujimura et al., 2007). In the past few decades, groundwater was heavily exploited due to drought and growth of the world’s economy and population (Llamas & Martínez-Santos, 2005). The world’s population is facing water crisis which is expected to worsen during the twenty-first century (Luczaj, 2016). In this respect, the main goal of the United Nations (UN) for sustainable development is safe drinking water; however, in many countries, the goal remains far off (Radelyuk et al., 2021). The long-term heavy exploitation of groundwater induces quantity and quality deterioration of the resource. Accordingly, the major challenge facing the world is overexploitation of groundwater as well as natural and anthropogenic contaminations (Luczaj, 2016). Changes in groundwater compositions also result from reactions occurring along flow path (e.g., leaching of surficial salts, water–rock interaction, and/or mixing with other sources) (Sami, 1992). Additionally, the extensive use of fertilizers for enhancing crop yields leads to groundwater contamination and deterioration (Bouzourra et al., 2015; El Alfy & Faraj, 2016; Milhome et al., 2015). Several studies were made to see the geogenic and anthropogenic effects on groundwater composition using several approaches (e.g., multivariate statistical analysis (Abu Salem et al., 2017, 2021; Panagopoulos et al., 2016; Reghunath et al., 2002; Srivastava et al., 2012), and hydrogeochemical modeling (Eissa et al., 2013; Hidalgo & Cruz-Sanjulián, 2001; Londoño et al., 2008; Mohamed et al., 2015)). The main aim of these studies is to infer the controls on the groundwater chemistry to confirm or preclude other processes (e.g., anthropogenic impacts, salt recycling, geogenic effects). Additionally, water quality index (WQI) is used for water quality assessment through the determination of physicochemical parameters of water to investigate the influence of natural and anthropogenic activities (WHO, 1997). It is one of the most effective tools to provide an assessment for water quality to the policy makers and environmentalists (Mohammed et al., 2022; USEPA, 2008). This index gives a single number expressing overall water quality status of a certain time and location (EPA, 2014).

Recently, Egypt underwent water shortage due to high population growth and expected lowering of the River Nile share due to the construction of the Grand Ethiopian Renaissance Dam (GERD) (Albadr, 2021). Subsequently, the increasing demands and water shortage are overcome by heavy exploitation of surface and groundwater that induces water quality problems. To unravel the processes controlling the composition and pollution sources of the groundwater in southwest of Beni Suef governorate, a comprehensive hydrogeochemical and multivariate statistical analysis were done. The use of statistical analysis in combination with geochemical tools helps reveal the different processes that control ionic compositions of groundwater, and to indicate the hydrogeochemical evolution as well as contamination sources (Steinhorst & Williams, 1985; Cloutier et al., 2008; Mohamed et al., 2015; Abu Salem et al., 2021; 2022). The integration between these methods helps infer the possible ionic sources for better water quality management.

**Fig. 1:**
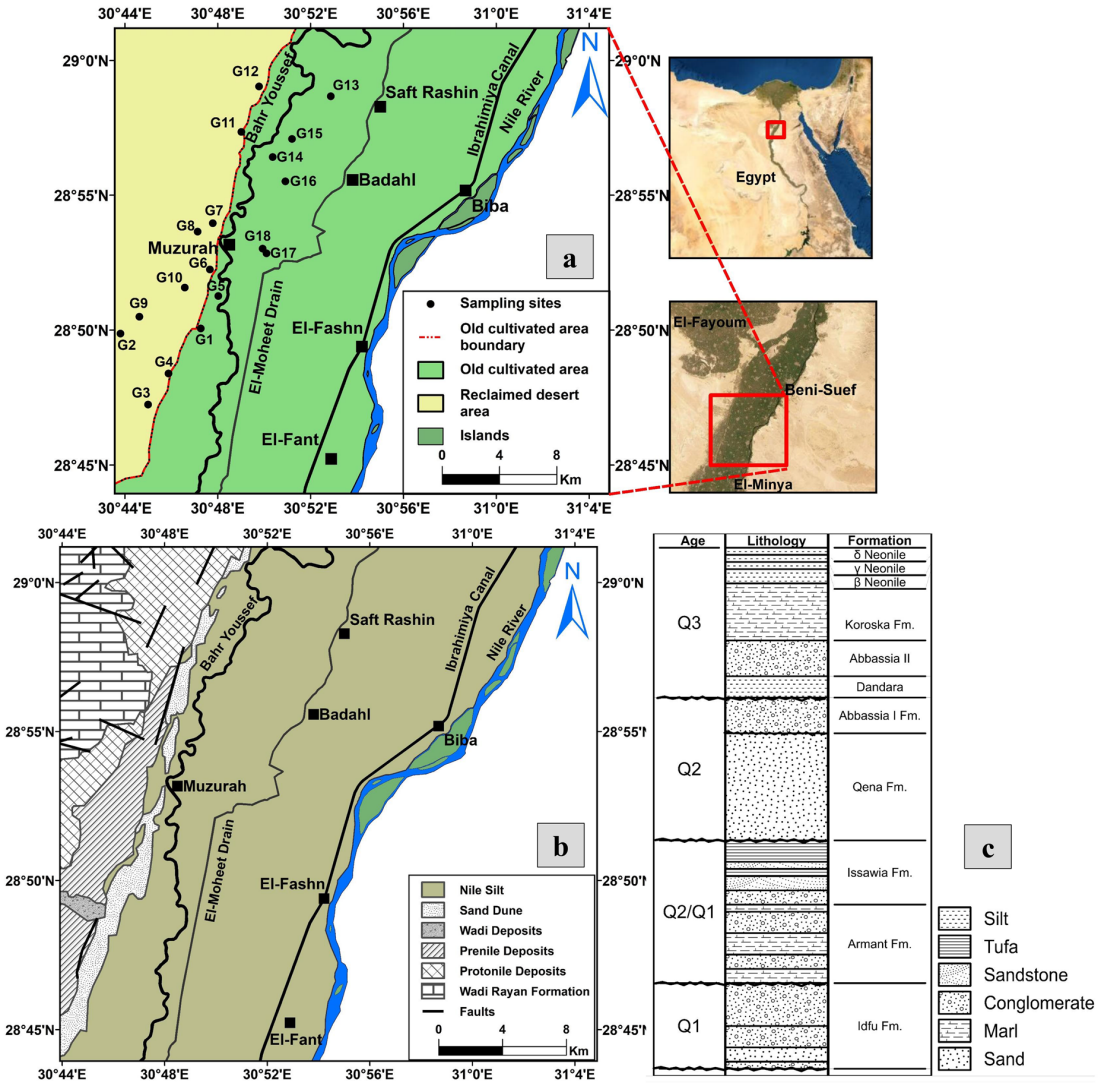
**a** Location map of Beni Suef in El Fashn District, Egypt. **b** Geological map of the study area (modified after CONOCO Coral et al., 1987). **c** Lithostratigraphic column of surface sediments based on measured sections along the Nile Valley banks (modified after Said, 1990)

In the study area, the main developmental activity is agriculture where the water is obtained from the River Nile, the Ibrahimiya canal, Bahr Youssef canal, irrigation canals, and drains in addition to groundwater (Fig. 1a). Groundwater is only used in areas distant from the surface water or in seasons of increased demand. Approximately, 92% of the Nile River water share for Beni Suef governorate is used for irrigation, 5% for drinking, and 3% for industry (EEAA, 2003). The irrigation is commonly achieved through flood techniques while drip irrigation is used in a limited extent. The flood irrigation and the lack of adequate drains in the western parts of the study area pose a substantial threat to groundwater due to the excess polluted agricultural return flow that could contaminate groundwater (Albadr, 2021). Moreover, waterlogging is dominant in some parts of the study area, resulting in soil amendment and may be abandonment (Albadr et al., 2021). Accordingly, the main objective of this study is the investigation of the hydrogeochemical properties of the Quaternary aquifer in Beni Suef area based on analyzing the groundwater samples collected during summer season in 2018. In addition, the water quality for different purposes is assessed.

## Study area

The investigated area is located in the western part of the Nile valley, in El-Fashn District southwest of Beni Suef Governorate, Egypt. It is located between latitudes 28°43′ and 29°01′N and longitudes 30°43′ and 31°02′E (Fig. 1a). It is also bounded from the east by the Nile River, from the west by the Western Desert, from the north by El-Faiyum Governorate, and from the south by El-Minya Governorate, with an area of about 890 km^2^. It is characterized by four distinctive surface water bodies: the River Nile and the Ibrahimiya canal in east, El-Moheet drain in the middle, and Bahr Youssef canal in the west. These water bodies run in the old cultivated area to the east of the newly reclaimed desert area (Fig. 1a).

Beni Suef area has an arid desert climate typical of Group BWh in the Köppen climate classification (Köppen, 1918; Peel et al., 2007). It is characterized by little precipitation, hot summer, and warm winter with cool nights.

Geomorphologically, the study area is classified regionally into four units which are young alluvial plains, fanglomerates, old alluvial plains, and calcareous plateau (Said, 1981).

The study area is covered by the Quaternary deposits that lie unconformably over the Pliocene and older sediments (Fig. 1b, c) (Attia, 1954; Hassan et al., 1978; Said, 1981, 1990). Several structural lineaments control the structural setting of the study area: the Red Sea and the Gulf of Suez (NW–SE) and the Syrian Arc system (NE-SW) (El Abd, 2015). Additionally, the Pleistocene deposits rest unconformably on the Eocene deposits in the surface exposures while they rest conformably on the Pliocene deposits in the subsurface. These deposits comprise the two main investigated aquifers: the Middle Pleistocene (the Prenile) aquifer and the Early Pleistocene (the Protonile) aquifer. According to Said (1990), the Pleistocene deposits are divided into three units on the basis of their texture, structural, and mineral composition to the following.

*The Early Pleistocene (the Protonile deposits, Q1)* is represented by Idfu Formation that is made up of gravels and sands of quartz and quartzite compositions and are embedded in a red-brown matrix of thickness exceeding 20 m (Said, 1975, 1981).

*Plio-Pleistocene (Paleo-Protonile Interval)* is characterized by two different formations belonging to the Early Pleistocene: an older Armant Formation which is made up of alternating beds of locally derived gravels cemented by tuffaceous materials, and fine-grained clastic rocks that are calcareous sandy, shaly, or phosphatic depending on the nearby source rock (Said, 1975, 1981). The Armant Formation is overlain by the Issawiya Formation that is composed of bedded and/or massive tufas associated with thick talus breccias deposited during high seismicity episode (Said, 1990).

*The Middle Pleistocene (The Prenile deposits, Q2)* is represented by Qena Formation that is made up of massive cross-bedded fluvial sands interbedded with dune sand, and is terminated by deposition of the Abbassia Formation which is composed of massive loosely consolidated gravels of polygenetic origin which are derived from uncovered basement of the Eastern Desert after the severance of connection between the Egyptian Nile with the Ethiopian Highlands (Said, 1981, 1990).

*The Late Pleistocene (The Neonile deposits, Q3)* is represented by the Korosko Formation which includes the deposits of two major Pluvials: the Saharan I and Saharan II, with two or more silts occurring in the midst of recessional deposits (Said, 1990). Butzer and Hansen (1968) described the Korosko Formation as sandy-marly unit forming basal sands and marls of Kom Ombo Nilotic succession followed by the deposition of Younger Neonile deposits (β, γ, δ deposits) that consist of massive structured silts with interfingering dune sand (Said, 1990).

*The Holocene Deposits* are formed of unconsolidated sediments that fill the study area and comprise the Nile silts (act as aquitard for the Prenile aquifer), the sand dunes, and the fanglomerates.

The Quaternary aquifer in the study area is characterized by high potentiality according to the Gheorghe classification (Albadr et al., 2021), where the transmissivity varies from about 769 to 4796 m^2^/day for the Early Pleistocene aquifer, and from 656 to 28,602 m^2^/day for the Middle Pleistocene aquifer (Albadr et al., 2021). Additionally, the Middle and the Early Pleistocene aquifers are hydraulically connected (El Sayed, 1993). The groundwater flow direction is from west to northeast direction following the general slope; however, a slight rise in groundwater levels is observed in the west due to application of flood irrigation technique (Albadr et al., 2021).

## Methodology

Eighteen groundwater samples were collected in duplicates in polypropylene bottles after pumping the wells for 15 min. Sampling locations were determined using GPS (Trimble Model Juno T41/5) (Fig. 1a). Temperature, pH, and electrical conductivity (EC) were measured in situ using calibrated instruments (HANNA HI 8314 membrane pH meter and ProLine B250 model Conductivity meter). The readings were taken after they were stabilized. A set of the duplicate samples (18 samples) were filtrated through 0.45-µm filter paper then acidified to pH ≤ 2 by ultra-pure HNO_3_ (1:1) for heavy metal analysis and kept at ≤ 6 °C according to Baird et al. (2017). The other set of the duplicate samples were analyzed for TDS and major elements. The TDS is measured by the gravimetric method while the major anions were measured using ion chromatography (IC- model DX-600, USA), with detection limit of SO_4_ < 0.5 and Cl < 0.2 mg/l. Major cations and heavy metals (Fe, Mn, Cu, Co, Ni, Zn, V, Sb, Pb, Se, Ni, Cr, Ba, Cd, Al, As, Sn) were measured by inductively coupled plasma-emission spectrometry (ICP-ES) with ultra-sonic Nebulizer USA (model Perkin Elmer Optima 3000, USA). The Nebulizer decreases the instrumental detection limits by 10% (Table S1). Analysis was performed in the National Water Research Centre (NWRC), central laboratory for environmental quality monitoring. The analyzed samples were treated statistically using IBM^®^ SPSS^®^ Statistics (Version 22) software. Finally, WQI computer code was used to evaluate water quality for drinking and domestic uses.

### Water quality evaluation

The water quality index of the Canadian Council of Minister of Environment (WQI_CCME_) is used to assess water quality for drinking and domestic uses in relation to contaminant characterization according to the Egyptian Health Authority guidelines (EHA, 2007).

#### Calculation of water quality index (WQI_CCME_)

This index comprises three factors calculated after the definition of water type, time, and variables. These calculations are explained as follows (CCME, 2001):

Scope (F1) represents the extent of water quality guideline non-compliance over the time period of interest.

$$\mathrm F1=\frac{\mathrm{Number\;of\;failed\;variables}}{\mathrm{Total\;number\;of\;variables}}\times100$$

Frequency (F2) represents the percentage of individual tests that do not meet objectives (failed tests).

$$\mathrm F2=\frac{\mathrm{Number\;of\;failed\;tests}}{\mathrm{Total\;number\;of\;tests}}\times100$$

Amplitude (F3) represents the amount by which the failed test values do not meet their objectives. This is calculated in three steps:

Excursion is the number of times by which an individual concentration is greater than (or less than, when the objective is a minimum) the objective:

$${\mathrm{Excursion}}_{\mathrm i}=\frac{\mathrm{Failed\;test\;value\;i}}{\mathrm{Guideline\;j}}-1$$

When the test value must not fall below the objective:

$${\mathrm{Excursion}}_{\mathrm i}=\frac{\mathrm{Guideline\;j}}{\mathrm{Failed\;test\;value\;i}}-1$$

Normalized sum of excursions (*nse*) is the collective amount by which individual tests are out of compliance. This is calculated by summing the excursions of individual tests from their objectives and dividing by the total number of tests (both those meeting objectives and those not meeting objectives).

$$nse=\frac{\sum \mathrm{excursions}}{\mathrm{Total \;number \;of \;tests}}$$

F3 is calculated by an asymptotic function that scales the normalized sum of the excursions from objectives to yield a range from 0 to 100.

$$\mathrm F3=\frac{nse}{0.01nse+0.01}$$

The WQI is then calculated as follows:

$$\mathrm{WQI}=100-\frac{\sqrt{{\mathrm F1}^2+{\mathrm F2}^2+{\mathrm F3}^2}}{1.732}$$

Once the WQI_CCME_ value has been calculated, water quality is ranked by relating it to one of the categories in Table S2.

Other parameters were used to assess the water quality for irrigation and for suitability for livestock and poultry (Tables S3–S5).

In this work, several software were used: Google Earth Pro v7.3.2.5776 was used to obtain the satellite image. Grapher Version 14.2.371 was used for drawing Piper diagram. Global Mapper v17.1.0 and ArcGIS 10.3 and 10.5 were used for designing different maps. AquaChem Version 2014.2 was applied for drawing Stiff diagrams and US Salinity. PHREEQC interactive 3.4.0–12,927 software was used for saturation indices and ion activity calculations (Parkhurst & Appelo, 2013).

## Results and discussions

### Statistical analysis

#### Descriptive statistics

The descriptive statistics of the variables of the analyzed samples were calculated and compared to the limits of the drinking water quality standards of the Environmental Protection Agency (EPA, 2018), the World Health Organization (WHO, 2011), and the Egyptian Health Authority (EHA, 2007) (Table 1).

**Table 1:** Descriptive statistical data of the analyzed groundwater samples. Element concentrations and TDS are in mg/L, EC is in µS/cm, and temperature is in degree Celsius. Guidelines for water quality of drinking water by EPA (2018), WHO (2011), and EHA (2007)

Parameter	Min	Max	Mean	S.D	EPA, 2018	WHO, 2011	EHA, 2007
pH	6.88	7.42	7.18	0.17	6.5–8.5	6.5–8.5	6.5–8.5
Temp	21.8	28.4	24.09	1.65	–	–	–
EC	608	7770	2702	1735	–	–	–
TDS	388	5056	1768	1143	500	1000	1000
HCO_3_^−^	175	660	344.3	101.8	–	–	–
Ca^2+^	42.18	669	288.8	209.1	–	–	–
K^+^	8	34	15.39	7.49	–	–	–
Mg^2+^	13.6	59.4	29.57	12.52	–	–	–
Na^+^	52	1067	272.5	224.3	200	200*	200
Cl^−^	21.55	1488	355.5	346.4	250	250*	250
PO_4_^3−^	0.1	11.37	1.26	2.81	–	–	–
SO_4_^2−^	52.86	1545	600.5	500.9	250	500**	250
Al	0.019	0.304	0.109	0.068	0.2	0.2	0.2
Ba	0.025	0.18	0.083	0.053	2	0.7	0.7
Cr	0.001	0.013	0.001	0.003	0.1	0.05	0.05
Co	0.0020	0.006	0.0035	0.0013	–	–	–
Cu	0.039	0.208	0.083	0.049	1.3	2	2
Fe	0.062	0.788	0.209	0.172	0.3	0.3	0.3
Mn	0.011	1.14	0.337	0.272	0.05	0.4	0.4
Ni	0.001	0.024	0.007	0.006	0.02	0.07	0.02
V	0.0025	0.015	0.0036	0.0031	–	–	–
Zn	0.005	0.422	0.052	0.094	5	3	3

Environmental Protection Agency (EPA, 2018)

World Health Organization (WHO, 2011)

Egyptian Health Authority (EHA, 2007)

*No health-based guideline is proposed, but a taste threshold exists

**No health-based guideline is proposed for SO_4_^2+^, the threshold is for taste, corrosion, and gastrointestinal effects resulted from higher SO_4_^2+^ levels

The pH values range from 6.88 to 7.42, displaying the range found in most natural waters (Hem, 1985) that lie within the desirable limits of the drinking water quality standards (Table 1). The TDS ranges from 388 to 5056 with a mean of 1768 mg/L. According to Todd (1980), the groundwater varies from fresh (less than 1000 mg/L) to brackish (1000–10,000 mg/L). Details about the minima, maxima, and means of the major cations (Ca^2+^, K^+^, Mg^2+^, Na^+^) indicate that only the maximum Na^+^ concentrations in the studied samples exceed the concentrations of the EPA (2018) standards for drinking by 4.34 folds (Table 1). On the other hand, the major anions that show higher limits compared to the EPA (2018) standards are SO_4_^2−^ and Cl^−^ with maxima exceeding the limits of EPA (2018) by 5.18 and 4.95 folds for SO_4_^2−^ and Cl^−^ respectively (Table 1). The PO_4_^3−^ concentrations range from 0.1 to 11.37 mg/L with a mean of 1.26 mg/L.

The concentrations of Mn, Fe, Al, and Ni have maxima that exceed the EPA (2018) limits by 21.8, 1.6, 0.52, and 0.2 folds, respectively (Table 1). The concentrations of Ba, Cr, Cu, and Zn lie within the drinking water quality standards, which are 0.025–0.18 mg/L, 0.001–0.013 mg/L, 0.039–0.208 mg/L, and 0.005–0.422 mg/L, respectively. Co and V have ranges 0.0015–0.006 mg/L and 0.0005–0.015 mg/L, respectively, where no health-based limits were set for them. Other trace elements (e.g., As, Cd, Sb, Se, Sn, and Pb) have concentrations below the detection limits 0.006, 0.002, 0.009, 0.007, 0.006, and 0.007, respectively.

#### Factor analysis

**Fig. 2:**
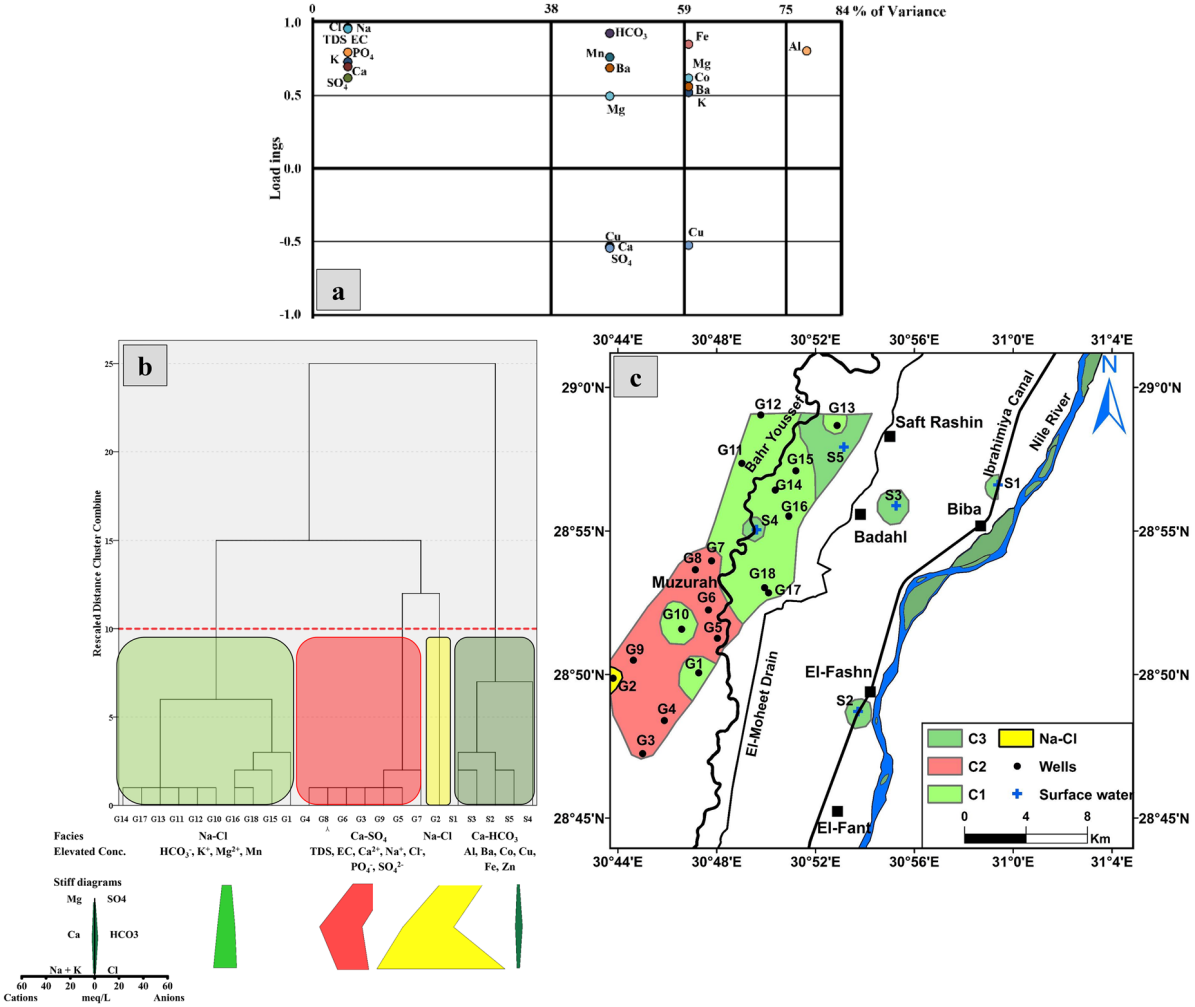
**a** Graphical presentation of the recognized factors for the studied groundwater samples. **b** Dendrogram based on hierarchical cluster analysis of the groundwater and surface water samples with Stiff graphs. Dotted red line defines the “phenon line,” which is chosen by the analyst to select number of groups or subgroups. **c** Spatial distribution map of clusters in the study area

Factor analysis is a multivariate statistical technique used to analyze interrelationships among large number of variables into a smaller set of dimensions (factors) with a minimum loss of information (Hair et al., 1992). The principal component analysis (PCA) was used to infer the processes controlling the concentration of ions in the studied groundwater samples. To determine the sampling adequacy and the feasibility of applying the PCA, the Kaiser–Meyer–Olkin (KMO) and Bartlett’s sphericity tests were applied first (Table 2) (Field, 2009). The best adequacy results were obtained by using sixteen variables (K^+^, Na^+^, Mg^2+^, Ca^2+^, Cl^−^, SO_4_^2−^, HCO_3_^−^, PO_4_^3−^, EC, TDS, Fe, Mn, Ba, Al, Co, and Cu). Accordingly, the KMO test result was 0.590 confirming the application of PCA in reducing the dimensionality of the dataset as there is inter-correlation between the variables. Additionally, the results from the Bartlett sphericity test (chi-square = 428.4; degree of freedom = 120; and *p* < 0.001 “ = zero”) confirm that there is common variance shared among the studied variables. Based on eigenvalues (more than one) and varimax rotation, four factors were distinguished to explain most of the variability with total cumulative variance of about 83.78% for the groundwater samples (Table 2; Fig. 2a).

**Table 2:** The Kaiser–Meyer–Olkin (KMO) and Bartlett’s sphericity tests and the principal component analysis (PCA) for the analyzed groundwater samples

Kaiser–Meyer–Olkin measure of sampling adequacy					0.59
Bartlett’s test of sphericity	Approx. chi-square				428.444
	df				120
	Sig	0			
Rotation sums of squared loadings	Total	6.04	3.39	2.59	1.39
	% of variance	37.78	21.17	16.16	8.67
	Cumulative %	37.78	58.95	75.11	83.78
		1	2	3	4
	Na^+^	**0.97**	0.1	−0.15	0.06
	EC	**0.96**	−0.19	−0.10	−0.13
	TDS	**0.96**	−0.20	−0.08	−0.14
	Cl^−^	**0.96**	0.04	0.17	0.15
	PO_4_^3−^	**0.8**	−0.10	−0.14	0.23
	K^+^	**0.73**	0.34	**0.52**	0.11
	Ca^2+^	**0.7**	−0.53	−0.12	−0.39
	SO_4_^2−^	**0.62**	−0.54	−0.30	−0.40
	HCO_3_^−^	0.06	**0.93**	−0.17	−0.08
	Mn	−0.06	**0.76**	0.02	−0.32
	Ba	−0.26	**0.69**	**0.56**	0.12
	Cu	0.24	−0.55	−0.53	0.2
	Fe	0	−0.27	**0.85**	0.08
	Co	−0.11	0.08	**0.62**	−0.08
	Mg	0.39	**0.50**	**0.62**	0.34
	Al	0.04	−0.24	−0.03	**0.81**

Bold entries significant loadings (more than 0.5)

Extraction method: principal component analysis

Rotation method: varimax with Kaiser normalization

*Factor 1* accounts for about 38% of the total variance. It is characterized by strong to moderate positive loadings on Na^+^, EC, TDS, Cl^−^, PO_4_^3−^, K^+^, Ca^2+^, and SO_4_^2−^ which are 0.97, 0.96, 0.96, 0.96, 0.80, 0.73, 0.70, and 0.62, respectively. This could be termed the salinization factor, which is characterized by water rock interaction and ion exchange processes.

*Factor 2* explains about 21% of the total variance, in which strong to moderate positive loadings were found on HCO_3_^−^ (0.93), Mn (0.76), Ba (0.69), and Mg^2+^ (0.50). Negative loadings on Ca^2+^ (− 0.53), SO_4_^2−^ (− 0.54), and Cu (− 0.55) also exist. This could be termed the anthropogenic/secondary enrichment factor. Fertilizers originated from carbonate and phosphatic fertilizers contain high content of Ba, Sr, and Rb (Senesi et al., 1983). These types of fertilizers could affect the composition of the groundwater.

*Factor 3* accounts for about 16% of the total variance, where strong to moderate positive loadings on Fe (0.85), Co (0.62), Mg^2+^ (0.62), Ba (0.56), and K^+^ (0.52) exist in addition to negative loading on Cu (− 0.53). This could be termed the secondary and the micro- nutrients fertilizers. The secondary nutrients include S, Mg, and Ca, where the micro-nutrients include Fe, Zn, and B. Both nutrients are essential to plant growth to attain crop productivity and profitability. Additionally, for animal nutrition, additional elements may be applied through fertilizers such as Co (essential for plants for N-fixing bacteria and blue green algae), Se, Cr, and V (Roy et al., 2006). Todd et al. (1976) recognized that secondary fertilizers are rich in Ca, Mg, S, B, Mn, Cu, Zn, Mo, Cl, Co, V, and Na.

*Factor 4* expresses about 9% of the total variance, in which a strong positive loading on Al (0.81) is apparent. This could be related to aluminum fertilizers such as Al_2_(SO_4_)_3_.

#### Hierarchical cluster analysis

The most applied method in statistics is the hierarchical cluster analysis (HCA) (Davis & Sampson, 1986) that is used in the classification of data (Abu Salem et al., 2021; Cloutier et al., 2008; Güler et al., 2002; Ribeiro & Macedo, 1995; Schot & Van der Wal, 1992; Steinhorst & Williams, 1985). The dataset was standardized before applying HCA to the *Z* scores to avoid misclassifications that arise from the orders of magnitude and variances of the variables. The HCA was then applied to the collected groundwater samples as well as some selected surface water samples (5 samples) from Melegy et al. (2014) (Fig. 2b) using eighteen variables (pH, K^+^, Na^+^, Mg^2+^, Ca^2+^, Cl^−^, SO_4_^2−^, HCO_3_^−^, PO_4_^3−^, EC, TDS, Fe, Mn, Ba, Al, Co, Cu, and Zn). The clustering was applied using the Ward method and squared Euclidean distance which minimize the sum of squared distance of an object to its cluster centroid (SPSS, 2013). Accordingly, three major clusters were defined (C1, C2, C3) in addition to an independent case (G2) using a phenon line at a linkage distance of 10. The mean concentrations of each cluster were presented by Stiff diagrams (Fig. 2b) to show the ionic dominance between groups. The means for each of the parameters produced by the HCA analysis were calculated and presented in Table S6.

The first cluster (C1) is characterized by Na-Cl facies with the prominent concentrations of HCO_3_^−^, K^+^, Mg^2+^, and Mn. The second cluster (C2) is characterized by Ca-SO_4_ facies with the highest concentrations of TDS, Ca^2+^, Na^+^, SO_4_^2−^, Cl^−^, PO_4_^3−^, and EC. The third cluster (C3) is characterized by Ca-HCO_3_ facies with the lowest concentrations of TDS and major ions albeit having the highest concentrations of Al, Ba, Co, Cu, Fe, and Zn. The independent case (G2) shows Na-Cl facies. The spatial distribution map of each cluster is shown in Fig. 2c.

### Hydrogeochemical analysis

#### Hydrogeochemical classification and water type

**Fig. 3:**
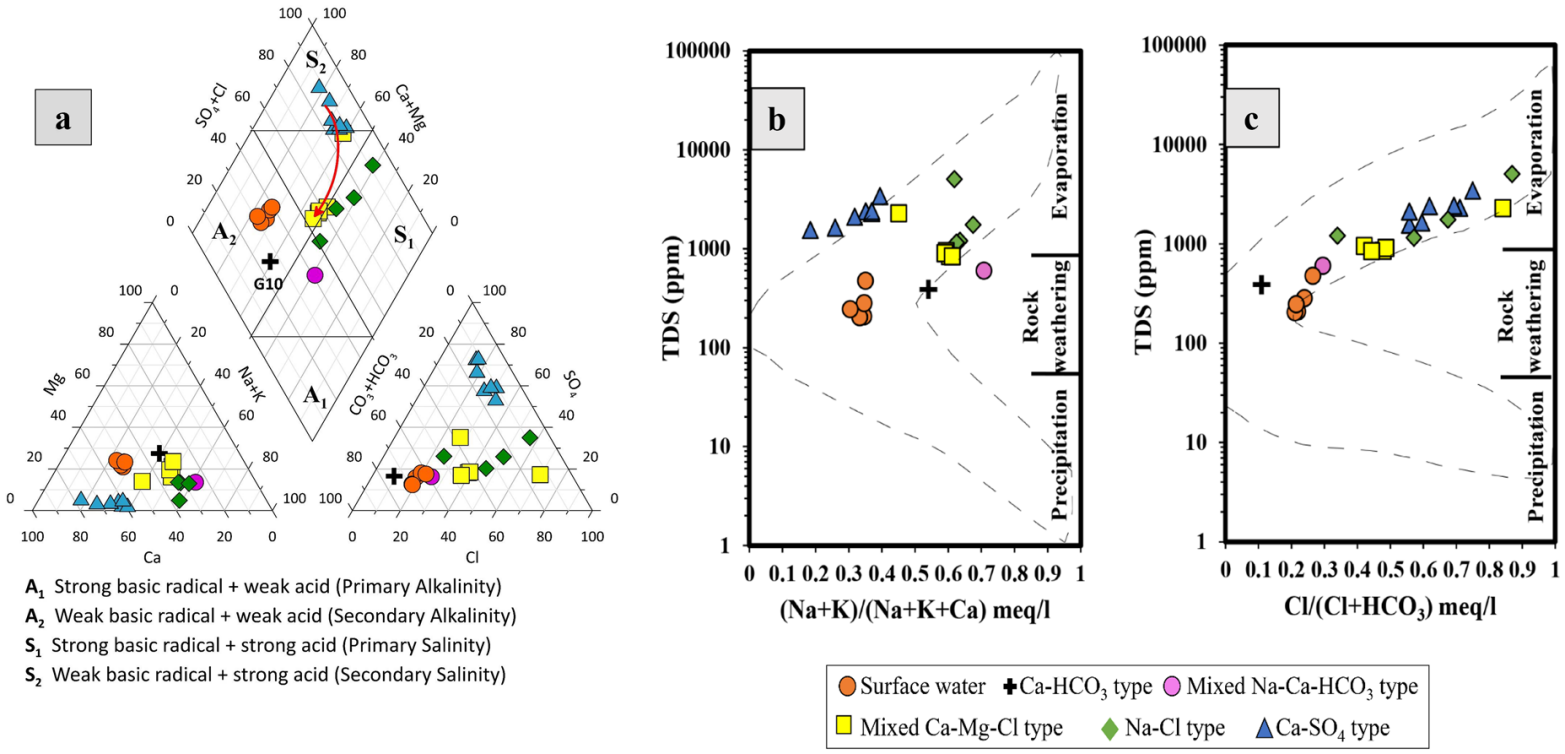
**a** Piper diagram (Piper, 1944), showing the different hydrogeochemical classes of the studied surface and groundwater samples. **b**, **c** Gibb’s diagram showing the hydrogeochemical processes affecting groundwater chemistry

The collected groundwater samples (18 samples) and the five reference surface water samples of Melegy et al. (2014) were plotted on the Piper diagram (Piper, 1944) to define the hydrogeochemical affinity of the studied samples. The plot of the water samples reveals five distinctive groundwater types: Ca-SO_4_ type (39%), mixed Ca–Mg–Cl type (27%), Na-Cl type (22%), mixed Na-Ca-HCO_3_ type (6%), and Ca-HCO_3_ type (6% of groundwater samples) in addition to the surface water samples that are represented by Ca-HCO_3_ type (Fig. 3a). The compositional change of the different classes indicates that along the major flow path (from southwest to northeast) the water composition changes from secondary salinity to mixed water and finally to primary salinity (refer to the red arrow in Fig. 3a).

#### Hydrogeochemical processes

The hydrogeochemical processes that control the chemistry of natural waters could be recognized using several relations between the ionic compositions. In the following context, the different processes and reactions will be established by drawing several *X*–*Y* relations using the major ion compositions as well as the saturation indices of common mineral phases.

The different hydrogeochemical processes such as evaporation, water–rock interaction, and precipitation could be inferred when plotting the water samples on the Gibbs diagram (e.g., Gibbs, 1970; Nosair et al., 2022). In this diagram, the plot of the ratio of either (Na^+^ + K^+^)/(Na^+^ + K^+^ + Ca^2+^) or Cl^−^/(Cl^−^  + HCO_3_^−^) as a function of TDS gives a characteristic nutshell that could be separated into three fields characterizing different processes that may affect the water composition (Fig. 3b, c). It shows that the main hydrogeochemical processes that control the composition of the studied water are evaporation (represented by Ca-SO_4_ type, Na-Cl type, and sample G1 of mixed Ca–Mg–Cl type) and rock-water interaction process (represented by Ca-HCO_3_ type, surface water, and the mixed Na-Ca-HCO_3_ type). Several samples of the mixed Ca–Mg–Cl type (G11, G12, G14, G17) show both evaporation and rock weathering processes (Fig. 3b, c).

- Ion exchange and reverse ion exchange processes

The ion exchange process expresses the Ca^2+^ and Mg^2+^ capture by Na-rich clays (Ettazarini, 2005) according to the reaction:

1 $${\mathrm{Ca}}^{2+}+2\mathrm{Na}-\mathrm{X}\to \mathrm{Ca}-\mathrm{X }+{2\mathrm{Na}}^{+}$$

2 $${\mathrm{Mg}}^{2+}+2\mathrm{Na}-\mathrm{X}\to \mathrm{Mg}-\mathrm{X }+{2\mathrm{Na}}^{+}$$

On the other hand, the reverse ion exchange is defined as the liberation of Ca and Mg in water according to the following equation:

3 $$\mathrm{Ca}-\mathrm{X }+ {2\mathrm{Na}}^{+} \leftrightarrow 2(\mathrm{Na}-\mathrm{X}) +{\mathrm{Ca}}^{2+}$$

**Fig. 4:**
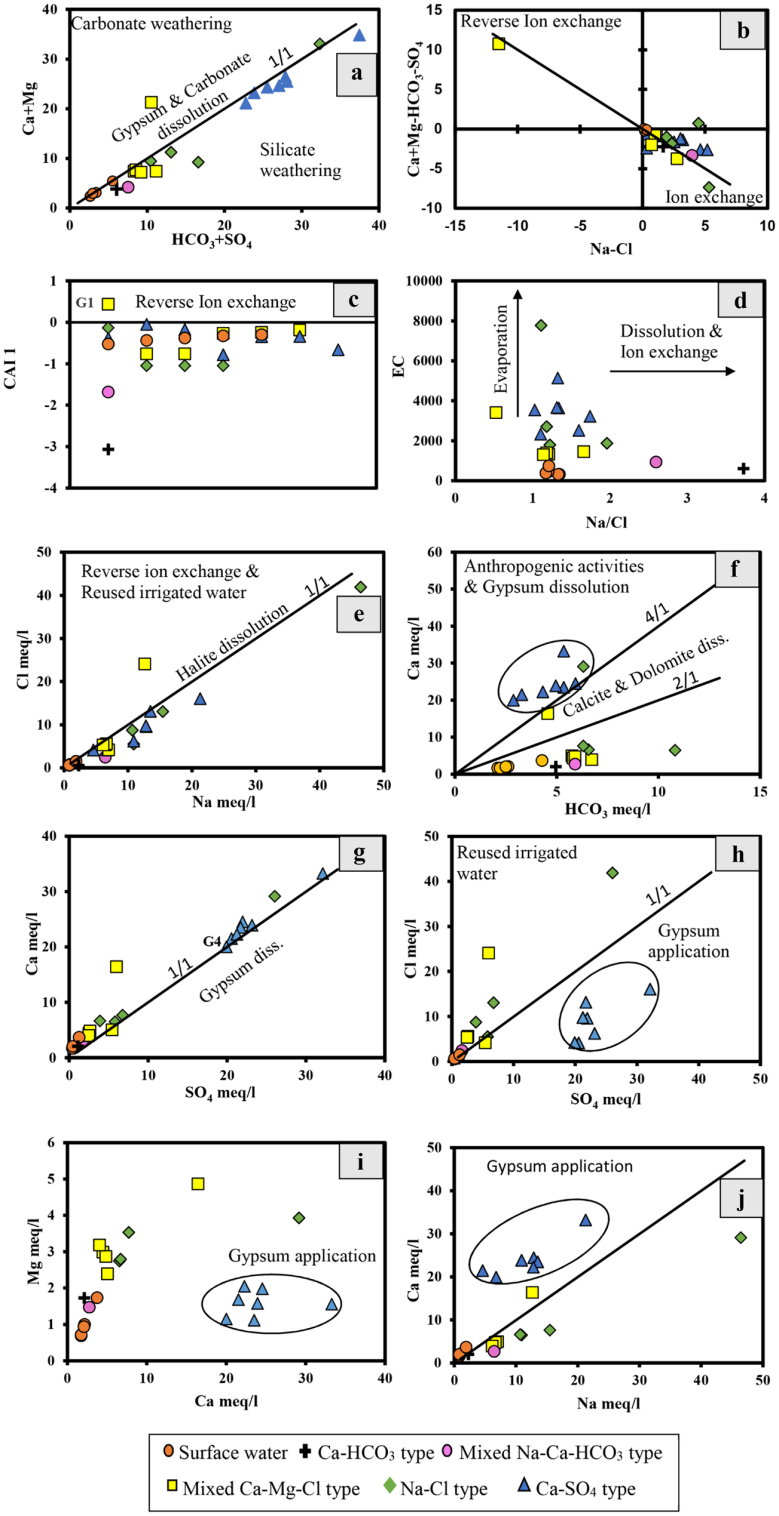
**a–j** Relationships between the different ions in the studied water samples

The plot of HCO_3_^−^  + SO_4_^2−^ versus Ca^2+^  + Mg^2+^ explains the possible processes that take place between the Ca^2+^ and Mg^2+^ and the Na-rich clays (Fig. 4a). Water is controlled by simultaneous dissolution of calcite, dolomite, and gypsum if they have a Ca^2+^ + Mg^2+^/SO_4_^2−^ + HCO_3_^−^ ratio of 1. The ratio less than unity indicates ion exchange process, while a ratio more than unity indicates a reverse ion exchange process (Cerling et al., 1989; Fisher & Mullican III, 1997). Most samples plot below the 1:1 line where an excess of HCO_3_^−^ + SO_4_^2−^ over Ca^2+^ + Mg^2+^. Only one sample (well G1) plots above the 1:1 (Fig. 4a).

The plot of Na^+^-Cl^−^ versus Ca^2+^ + Mg^2+^-HCO_3_^−^-SO_4_^2−^ is used to study the cationic exchange process, where the sample distribution along straight line with slope (− 1) indicates the existence of reverse ion exchange (Fisher & Mullican III, 1997; Subramani et al., 2010; Glover et al., 2012; Sajil Kumar & James, 2016; Karunanidhi et al., 2020). Only one sample (G1) exists in the reverse ion exchange zone while the rest of the samples are plotted in the ion exchange zone (Fig. 4b).

The chloro-alkaline indices also provide a valuable information about base exchange processes between groundwater and the aquifer sediments. It was assumed by Schoeller (1977) and has been used by several workers to identify the ion exchange processes controlling the groundwater chemistry (Aghazadeh & Mogaddam, 2011; Toumi et al., 2015; Zhu et al., 2007). The chloro-alkaline index (1) (CAI 1) is calculated using the following formula:

$$\mathrm{CAI }1= [{\mathrm{Cl}}^{-}- ({\mathrm{Na}}^{+}+{\mathrm{K}}^{+})]/{\mathrm{Cl}}^{-}$$

The CAI 1 shows a positive value when there is an exchange between Na^+^ and K^+^ in water with Ca^2+^ and Mg^2+^ in the rocks, while a negative index value indicates the reverse. Most of the water samples have a negative index value indicating ion exchange processes (Fig. 4c).

(b) Evaporation

The evaporation process is the most common process in arid and semi-arid conditions especially when groundwater has a shallow water level. Evaporation increases the concentration of TDS while Na^+^/Cl^−^ ratio remains constant (Jankowski & Acworth, 1997; Subramani et al., 2010). A scatter diagram of EC and Na^+^/Cl^−^ ratio indicates the occurrence of evaporation process while the Na^+^/Cl^−^ ratio tends to be constant with increasing salinity (Fig. 4d). When the Na^+^/Cl^−^ ratio is more than 1 at lower salinities, this indicates other sources of Na rather than a meteoric source (Nkotagu, 1996). The diagram shows that the surface water and the groundwater of the types Ca-HCO_3_, mixed Na-Ca-HCO_3_, and mixed Ca–Mg–Cl exhibit low salinity over a wide range of Na^+^/Cl^−^ ratio while the rest of the samples show high salinity with low Na^+^/Cl^−^ ratio (Fig. 4d). Accordingly, evaporation processes are confirmed where the EC of most of the studied samples increases under nearly constant Na^+^/Cl^−^ ratio, while the surface water expressing lower salinity with low Na^+^/Cl^−^ ratio (Fig. 4d).

Furthermore, a scatter diagram of Cl^−^ versus Na^+^ is used to indicate whether the groundwater is controlled by halite dissolution, reverse ion exchange, or silicate weathering (Fig. 4e) (Loni et al., 2015). Halite dissolution characterizes water samples that plot along the 1:1 line, while reverse ion exchange and possibly the irrigation return flow or sewage contamination characterize samples that plot above the 1:1 line (Jacks et al., 1999; Loni et al., 2015; Srinivasamoorthy et al., 2008). Conversely, silicate weathering or ion exchange reactions are represented by samples that plot below the 1:1 line (Fig. 4e). The collected groundwater samples show only one sample of Ca-SO_4_ type (G4) corresponds to the 1:1 line and one sample of mixed Ca–Mg–Cl type (G1) that plots above the 1:1 line where Cl^−^ is in excess. The rest of the water samples show excessive Na^+^ concentrations over than that of Cl^−^ (Fig. 4e).

The plot of SO_4_^2−^ and Ca^2+^ gives insights about gypsum dissolution when the samples plot along the 1:1 line (Fig. 4g). The collected groundwater samples show only one sample of Ca-SO_4_ type (G9) that corresponds to the 1:1 line (Fig. 4g) while the rest of the samples show excessive Ca^2+^ over SO_4_^2−^. The increase of Ca over SO_4_ indicates desorption of Ca^2+^ into water by reverse ion exchange process (Ettazarini, 2005).

(c): Silicate and carbonate weathering

Silicate and carbonate weathering are the most hydrogeochemical processes that contribute Na^+^, K^+^, Ca^2+^, and Mg^2+^ to the groundwater. The plot of HCO_3_^−^ + SO_4_^2−^ versus Ca^2+^ + Mg^2+^ is used to investigate these processes. A ratio of less than 1 indicates the dominance of silicate weathering over carbonate weathering whereas a ratio higher than 1 indicates the reverse (Kumar et al., 2009). The majority of the studied water samples show the effect of silicate weathering (Fig. 4a). The plot of Cl^−^ versus Na^+^ further reveals the dominance of silicate weathering or ion exchange process where Na^+^ exceeds Cl^−^ (Fig. 4e). Additionally, the relation of HCO_3_^−^ and Ca^2+^ is used to study the dominance of carbonate weathering in the aquifer (Fig. 4f). The plot of samples between the lines 1:4 and 1:2 indicates the dominance of calcite and dolomite dissolutions (carbonate weathering), whereas the plotting above the 1:4 line indicates excess of calcium over than expected from calcite—dolomite dissolution and could be attributed to Ca^2+^ and Mg^2+^ exchange by Na^+^ or K^+^ or due to gypsum dissolution according to the reactions (3, 4, and 5), or due to anthropogenic activities. Conversely, the plotting below the 1:2 line could be attributed to biological respiration or atmospheric CO_2_ dissolution according to reaction (6). The collected groundwater samples show that mostly the Ca-SO_4_ water type plots above the 1:4 line having excess Ca^2+^ over than expected from calcite/dolomite dissolution and could be attributed to Ca^2+^ and Mg^2+^ exchange by Na^+^ or K^+^ in clays or due to gypsum dissolution (Fig. 4f). However, gypsum dissolution cannot interpret the excess calcium content as all the analyzed water samples plot above the 1:1 line that represents equivalent ratios of calcium and sulfate (Fig. 4g), whereas the rest of them show increasing content of HCO_3_^−^ that possibly could be attributed to biological respiration or atmospheric CO_2_ dissolution (reaction 6, Fig. 4f).

4 $$\left(\mathrm{Mg}\right)-\mathrm{EX }+ {2{\mathrm{Na}}_{\left(\mathrm{aq}.\right)}}^{+}\leftrightarrow 2(\mathrm{Na}-\mathrm{EX})+{\mathrm{Mg}}^{2+}$$

5 $${\mathrm{CaSO}}_{4}.2{\mathrm{H}}_{2}\mathrm{O }\leftrightarrow {{\mathrm{Ca}}_{\left(\mathrm{aq}.\right)}}^{2+}+{{{\mathrm{SO}}_{4}}_{\left(\mathrm{aq}.\right)}}^{2-}+2{\mathrm{H}}_{2}\mathrm{O}$$

6 $${\mathrm{CO}}_{2}+ {\mathrm{H}}_{2}\mathrm{O }\leftrightarrow {\mathrm{H}}_{2}{\mathrm{CO}}_{3}\leftrightarrow {\mathrm{H}}^{+}+{{\mathrm{HCO}}_{3}}^{-}$$

(d): Anthropogenic activities

The anthropogenic impacts on the groundwater could be deciphered using the relation between the Cl^−^ and SO_4_^2−^ (Subramani et al., 2010), where the increase in Cl^−^ over SO_4_^2−^ could be attributed to the reuse of irrigation water and/or sewage contamination (Fig. 4h). Conversely, the increase in SO_4_^2−^ over Cl^−^ in cultivated lands is mainly attributed to the gypsum application in soil amendment (Fig. 4h) (Subramani et al., 2010). Additionally, the plot of Mg^2+^ versus Ca^2+^, and Ca^2+^ versus Na^+^ where there is excess of Ca^2+^ over both Mg^2+^ and Na^+^ may be related to gypsum applications in cultivation (Fig. 4i, j). The plot of the Ca-SO_4_ water type below the 1:1 line indicates excessive SO_4_^2−^ over Cl^−^ while the plot of the groundwater samples of the mixed Ca–Mg–Cl type (G1) and Na-Cl water type (G2) above the 1:1 line indicates excessive Cl^−^ over SO_4_^2−^ (Fig. 4h). Additionally, the Ca-SO_4_ water type shows excessive Ca^2+^ over both Mg^2+^ and Na^+^ (Fig. 4i, j). Therefore, the studied water samples show the effect of the reuse of irrigation water and gypsum application causing increased salinity.

#### Saturation indices and clay mineral stability fields

##### Saturation indices (SI)

The saturation index is used to determine if an equilibrium exists between the water and the solid phase(s) it encounters, and to recognize mineral dissolution and precipitation processes in aquifers (Redwan et al., 2016). The saturation indices of the water samples were calculated on the basis of the following equation:

7 $$\mathrm{SI}=\mathrm{ Log \;IAP}/K\mathrm{sp}$$

where IAP is the ion activity product and *K*_sp_ is the solubility product constant. The assessment of SI might be helpful for understanding the chemical processes occurring between groundwater and aquifer material, and determining the origin of dissolved ions in groundwater (Kumar & Singh, 2015).

**Fig. 5:**
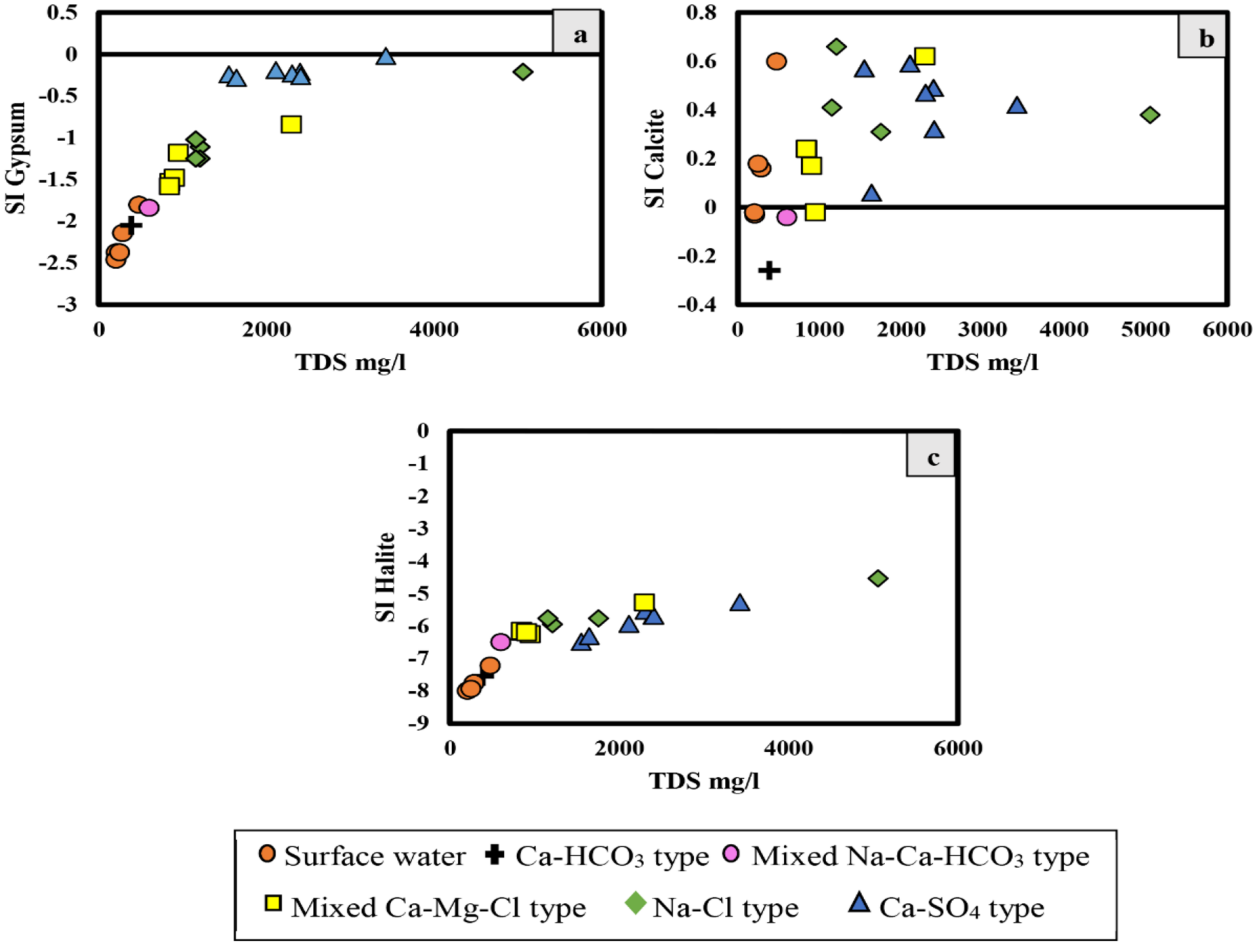
**a–c** Relationships between the saturation indices and the TDS in mg/L for selected mineral phases

The SI_Gypsum_ increases with increasing salinity changing from undersaturation to near saturation (Fig. 5a). No clear trend could be concluded from the relation of the SI_Calcite_ and TDS (Fig. 5b); however, it is oversaturated. Furthermore, a plot of SI_Halite_ versus TDS shows progressive increase in the SI_Halite_, although being undersaturated (Fig. 5c).

##### Clay minerals stability

Ion activities were calculated using PHREEQC interactive 3.4.0–12,927 software to provide more information about the clay mineral stability. The mineral stability diagrams are used to study the hydrogeochemical evolution of groundwater (Drever & Smith, 1978).

**Fig. 6:**
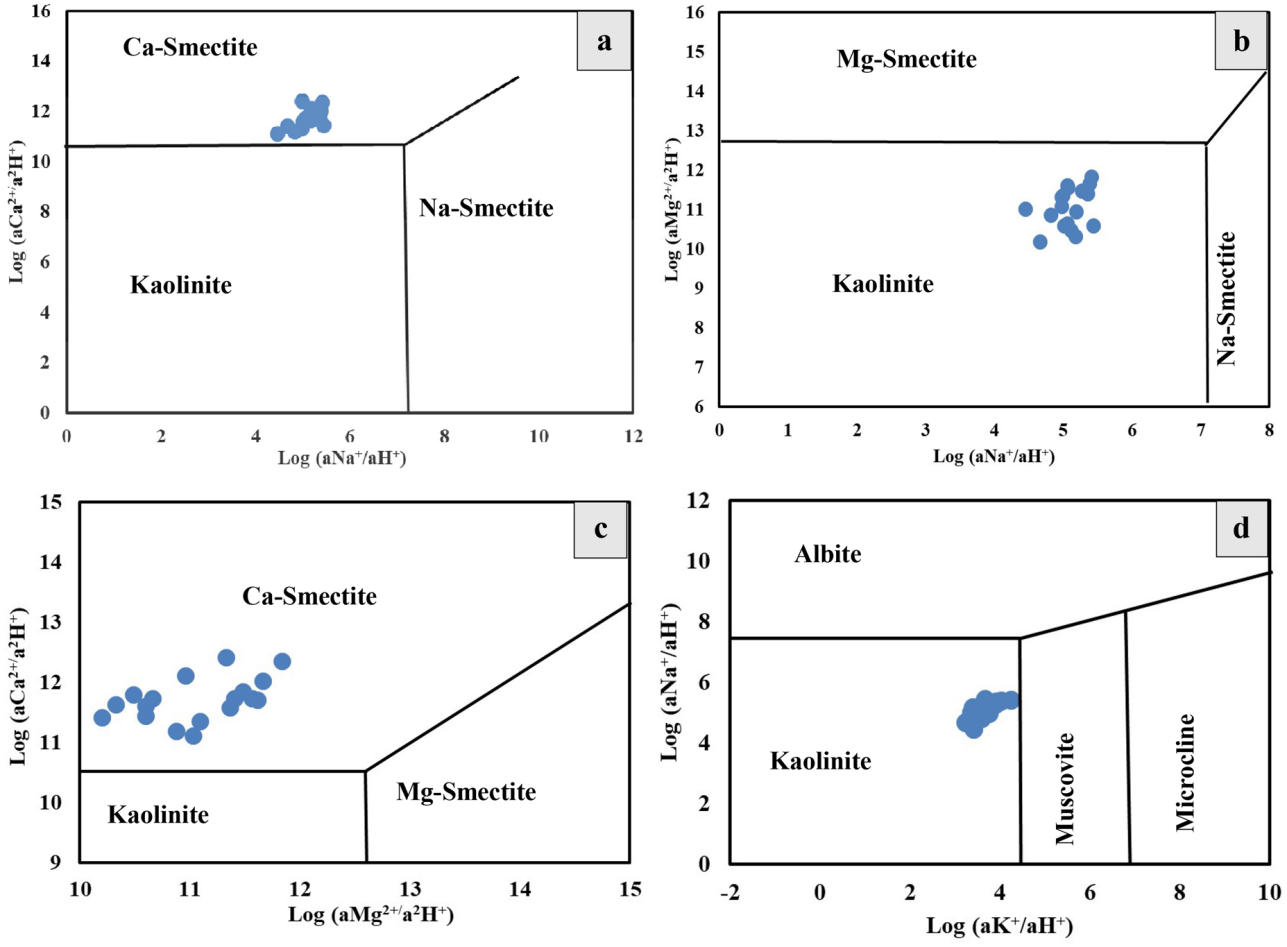
**a–d** Clay mineral stability diagrams for the analyzed groundwater samples

Scatter plots of log activities of Ca^2+^/(H^+^)^2^ and Na^+^/H^+^, and log activities of Ca^2+^/(H^+^)^2−^ and Mg^2+^/(H^+^)^2^ show that all the studied groundwater samples fall in the Ca-smectite field (Fig. 6a, c) and in the kaolinite field in a plots of both log activities of Mg^2+^/(H^+^)^2^ and Na^+^/H^+^, and log activities of Na^+^/H^+^ and K^+^/H^+^ (Fig. 6b, d). Therefore, the equilibrium with Ca-smectite and kaolinite is one of the main processes that control the water chemistry, where kaolinite represents a common weathering product of feldspar and other silicate minerals (Rajmohan & Elango, 2004). So, the major geochemical reaction controlling the chemistry of groundwater could be written as reaction 8:

8 $$6\;\mathrm{ Ca}{\mathrm{Al}}_{2}{\mathrm{SiO}}_{10}{\left(\mathrm{OH}\right)}_{2} (\mathrm{Ca}-\mathrm{Smectite})+2{\mathrm{H}}^{+}+23{\mathrm{H}}_{2}\mathrm{O}\leftrightarrow 7 {\mathrm{Al}}_{2}{\mathrm{Si}}_{2}{\mathrm{O}}_{5}{\left(\mathrm{OH}\right)}_{4}(\mathrm{Kaolinite})+{\mathrm{Ca}}^{2+}+8{\mathrm{H}}_{4}{\mathrm{SiO}}_{4}$$

### Geochemical modeling

**Fig. 7:**
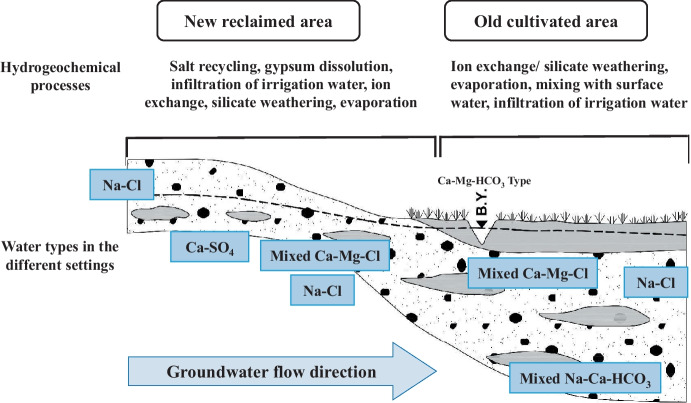
Schematic cross-section showing the suggested hydrogeochemical process controlling the groundwater composition in the study area

Based on the previous discussion, the hydrogeochemical processes controlling the groundwater chemistry in the study area are mainly affected by the groundwater occurrence either to the east (the old cultivation areas) or to the west (the new reclaimed area) of Bahr Youssef Canal (Fig. 7). Due to lacking surface water resources in the new reclaimed lands, the hydrogeochemical processes affecting the groundwater are the silicate weathering, and the ion exchange process with the aquifer materials, in addition to the infiltration of irrigation water that is enriched in Na^+^, Ca^2+^, and SO_4_^2−^ (Fig. 4 a, b, e, f, g, h, i, j). Additionally, in the new reclaimed area, the poor casing of domestic wells, the fertilizer application, and flooding irrigation system aid in aquifer contamination and salt recycling into the aquifer where the aquitard is diminishing. These combined processes result in Na-Cl and Ca-SO_4_ water types dominating the new reclaimed lands.

These areas especially west El Fashn are characterized by major soil problems such as low moisture capacity, low organic matter content, and the presence of sodium chloride and gypsum contamination (WMP, 1984). Accordingly, careful use of fertilizers in this area should be considered to overcome the adverse impact on groundwater quality.

In the old reclaimed lands, several surface water canals dominate representing the major recharge to the groundwater in addition to the infiltrated irrigation water. The mixed Ca–Mg–Cl and the Na-Cl water types characterize the groundwater in this area where the dominant reactions are ion exchange, silicate weathering, gypsum applications in soil, and evaporation (Fig. 4a, b, c, d, e, f).

Two types of wells characterize the old cultivated lands: shallow and deep wells. In the shallow wells (G14, G17), Ca–Mg–Cl type may result from mixing of infiltrated surface water of Bahr Youssef through the eastern bank with the infiltrated irrigation water. This water type in some sites could be influenced by evaporation process of surface water that is infiltrated to the aquifer (Figs. 3 and 4d). Silicate weathering or ion exchange process are additional processes that form the Na-Cl water type (Fig. 4a, b, c, d, e). In the deep well as in well G13 (well depth 100.9 m), mixed Na-Ca-HCO_3_ type may be related to ion exchange process and/or silicate weathering (Fig. 4a, b, c, d, e). The old cultivated lands are characterized by waterlogging phenomena that further deteriorate the soil where salt recycling into the aquifer may occur.

### Water quality evaluation

#### Assessment of water quality for drinking and domestic purposes

**Fig. 8:**
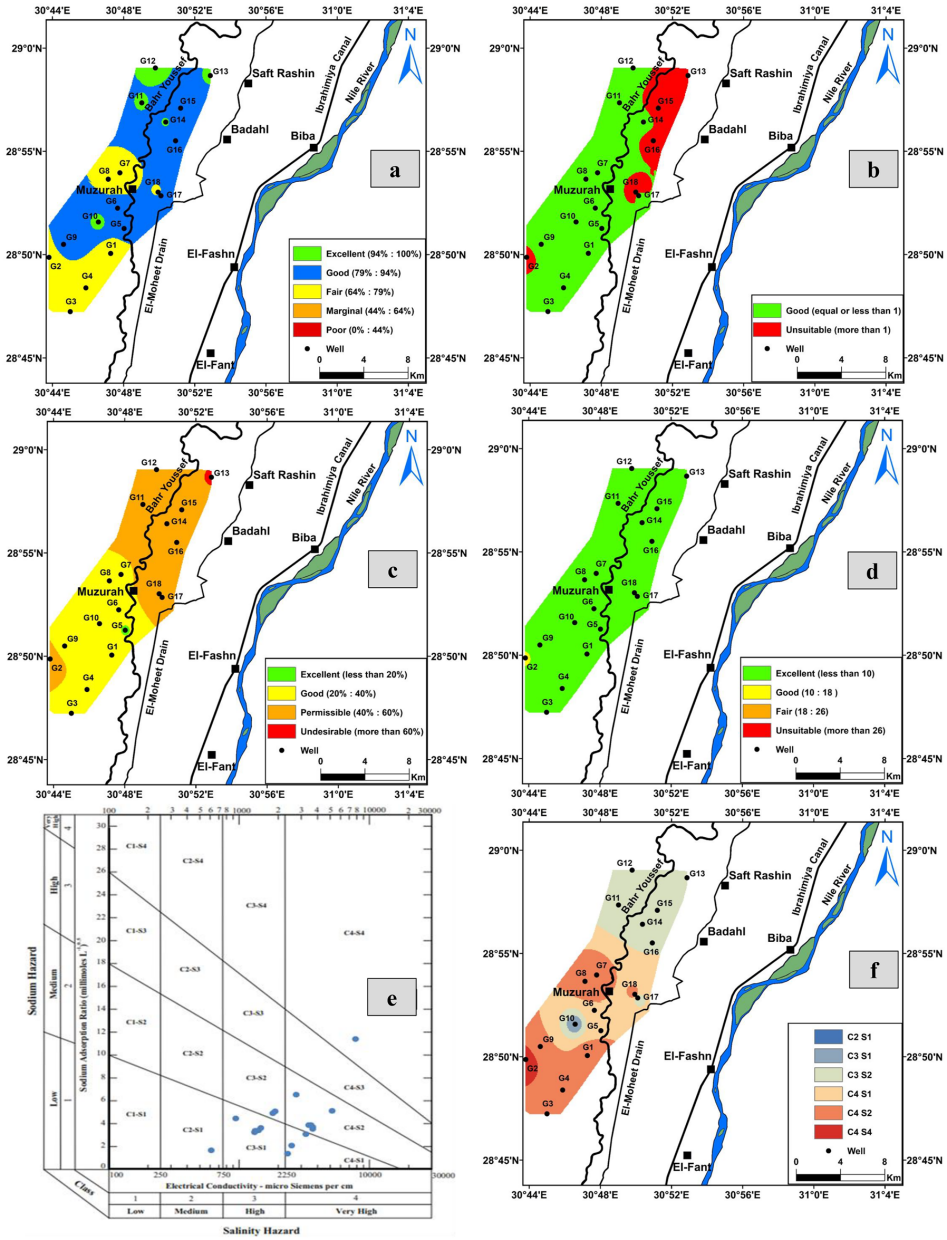
Distribution map of **a** WQI_CCME_ for the groundwater samples, **b** the groundwater suitability for agricultural uses based on Kelly’s ratio, **c** the groundwater suitability for agricultural purpose based on sodium percent, **d** the groundwater quality for irrigation purpose based on SAR ratio, **e** US Salinity diagram, and **f** distribution map of water quality classification for irrigation based on USSL (1954)

The Canadian Council of Minister of Environment (WQI_CCME_) is used to assess water quality of the studied water samples for drinking and domestic uses according to the Egyptian health authority guidelines. This index ranges from zero (poor quality) to 100 (excellent quality). The groundwater samples of the study area show a wide variation in quality ranks from fair (G2 of 65.08 index value) to excellent (G10 and G12 display an index value of 100) (Fig. 8a).

#### Assessment of water quality for irrigation purposes

##### Kelly’s ratio (KR)

This ratio is one of the parameters used to evaluate water suitability for irrigation. It is calculated using Eq. (9) as follows (Kelly, 1940):

9 $$\mathrm{KR}=\mathrm{Na}^+/(\mathrm{Ca}^{2+}+\mathrm{Mg}^{2+})\;\mathrm{meq}/\mathrm L$$

A ratio equal to or less than 1 indicates a good quality of water for irrigation, while a ratio more than 1 means the unsuitability of water for irrigation due to high sodium content (USSL Staff, 1954). According to KR, most of the analyzed groundwater samples show good quality for irrigation whereas few samples (G2, G13, G15, G16, G18) are unsuitable (Fig. 8b; Table S3).

#### Sodium percent (Na%)

Sodium percent (Na%) is used for the evaluation of the water quality for agricultural purposes (Wilcox, 1948). It is calculated by the using Eq. (10):

10 $$\mathrm N\mathrm a\%=\lbrack\frac{\mathrm{Na}^++\mathrm K^+}{\mathrm{Ca}^{2+}+\mathrm{Mg}^{2+}+\mathrm{Na}^++\mathrm K^+}\rbrack\times100\;\mathrm{meq}/\mathrm L$$

The water quality classes based on the Na% are given in Table S3, where the acceptable sodium content in water usually ranges from 0 to 40 meq/L (Ayers & Westcot, 1985). High sodium percent in irrigation water poses substantial hazards to plant growth as well as soil permeability reduction (Joshi et al., 2009). The groundwater with high sodium content will enhance the exchange reaction between soil and water, affecting the soil permeability and texture making it hard to plough and unsuitable for plant growth (Keesari et al., 2014).

The study area shows a wide variation in sodium percent values for groundwater samples where they range from 17.27 to 61.18%, indicating good to permissible water quality for irrigation except two samples; one of them exhibits excellent quality (G5 of Na% ~ 17.27%) and the other displays undesirable quality for agriculture (G13 of Na% ~ 61.18%) (Fig. 8c).

##### Sodium adsorption ratio (SAR)

The sodium adsorption ratio is a measurement of the sodium content or the alkali hazard that estimates the suitability degree of groundwater for irrigation purposes. The SAR ratio is calculated from Eq. (11):

11 $$\mathrm{SAR}=\mathrm{Na}^+/(\surd(\mathrm{Ca}^{2+}+\mathrm{Mg}^{2+})/2)\;\mathrm{meq}/\mathrm L$$

The SAR ratio is an important guideline due to its direct relation to the sodium adsorption by soil (Rao, 2006). The different quality classes for SAR are given in Table S3. All groundwater samples of the study area show excellent quality for irrigation uses according to the SAR classification, but only one sample (G2) exhibits good quality (Fig. 8d).

The US Salinity Laboratory staff (USSL Staff, 1954) is also used to understand the effect of both salinity hazard (expressed in term of EC) and sodium hazard (expressed in term of SAR) on the soil. The US Salinity diagram divides the water into several classes as C1, C2, C3, and C4, based on the salinity hazard, and S1, S2, S3, and S4 based on the sodium hazard (Fig. 8e). Zaman et al. (2018) discussed the properties and interpretation of each class (Table S4).

Groundwater samples of the study area fall in six sectors which are C2S1 (medium salinity with low sodium hazard), C3S1 (high salinity with low sodium hazard), C3S2 (high salinity with medium sodium hazard), C4S1 (very high salinity with low sodium hazard), C4S2 (very high salinity with medium sodium hazard), and C4S4 (very high salinity with very high sodium hazard) (Fig. 8f).

##### Residual sodium carbonate (RSC)

It represents an empirical parameter for predicting the additional alkalinity hazard associated with CaCO_3_ and MgCO_3_ (Eaton, 1950). It is calculated using Eq. (12):

12 $$\mathrm{RSC}=({\mathrm{CO}}_3^{2-}+{\mathrm{HCO}}_3^-)-(\mathrm{Ca}^{2+}+\mathrm{Mg}^{2+})\;\mathrm{meq}/\mathrm L$$

Eaton (1950) and Wilcox et al. (1954) classified the water quality for agricultural purposes based on RSC (Table S3). Accordingly, all groundwater samples are safe for irrigation uses except two samples (G13, G15) that are marginal for agricultural uses.

##### Magnesium ratio (MR)

Water can be classified as being unsuitable for irrigation when magnesium ratio is greater than 50% (Paliwal, 1972). This ratio is determined using Eq. (13):

13 $$\mathrm{MR}=[\frac{{\mathrm{Mg}}^{2+}}{{\mathrm{Ca}}^{2+}+{\mathrm{Mg}}^{2+}}]\times 100$$

The soil quality is affected by high magnesium content in water reducing the crop yield due the resultant alkaline nature of soil (Kumar et al., 2007). According to the MR classification, all groundwater samples are suitable for irrigation purpose.

#### Corrosivity ratio (CR)

The safety of groundwater to be transported through pipes is estimated by the corrosivity ratio which is calculated by the following expression:

14 $$\mathrm{CR}= ^{\left[{}^{{\mathrm{Cl}}^{-}}/_{35.5}+2\left({}^{{{\mathrm{SO}}_{4}}^{2-}}/_{96}\right)\right]}/_{2\left[{}^{{{\mathrm{HCO}}_{3}}^{-}+{{\mathrm{CO}}_{3}}^{2-}}/_{100}\right]}$$

Raman (1985) classified water into safe and unsafe according to the corrosivity ratio. A groundwater is safe for transport through pipes when the ratio is less than 1, whereas it is unsafe when it has a value more than 1, indicating corrosive nature (Tripathi et al., 2012). All groundwater samples are unsafe or corrosive on the basis of CR except samples G10 (0.28), G13 (0.57), G15 (0.86), and G17 (0.86).

**Fig. 9:**
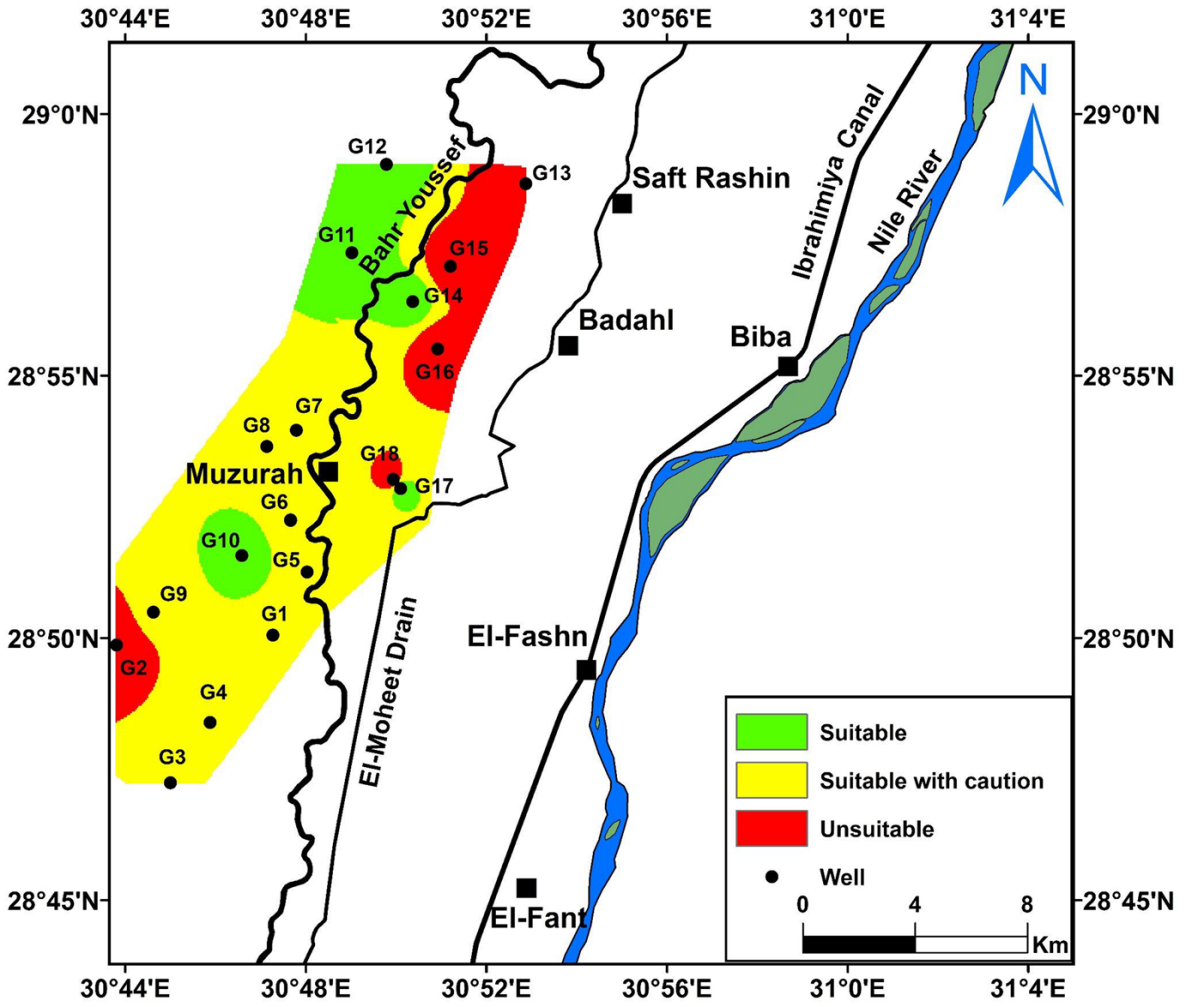
The suitability water map for irrigation based on KR, SAR, Na%, RSC, Mn, and USSL

Accordingly, the groundwater quality evaluated for the different purposes indicates that the water quality varies from fair to excellent for the drinking purposes, where the best water is located in the northern and central parts of the study area. Additionally, it is suitable for irrigation in the northwest and western parts of the study area. However, in the north, central, and northeastern parts, care should be considered because of increased KR, Na%, and unsuitable US Salinity classes, especially in samples G2, G13, G15, G16, and G18 (Fig. 9; Table S5). Singh (2000) noted that some high salinity classes on the US Salinity (e.g., C3S1, C3S2, C4S1, and C4S2) are suitable for plants with good salt tolerance but its suitability limits to soil with restricted drainage. C4S1 class is used in irrigation under certain conditions of high soil permeability, good leaching plants, and high tolerance to salts, in addition to chemical amendments (Hedjal et al., 2018). On the basis of CR for transport of groundwater through pipes, all groundwater samples are unsafe or corrosive except samples G10, G13, G15, and G17. So, it is favored to use corrosion resistance pipes for transporting these waters.

## Conclusions and recommendations

This study combines the use of multivariate statistical analysis and hydrogeochemical modeling to investigate the processes controlling the composition of a shallow aquifer where increased pumping rates and anthropogenic impacts on the groundwater were expected. The study area is characterized by the presence of surface water and groundwater that are used to provide the needed water for domestic and agricultural purposes. Stresses on the resource arise from extensive pumping, flood irrigation, and extensive use of fertilizers and pesticides. Groundwater is classified based on multivariate statistical analysis into three major clusters (C1, C2, C3) that differ in water type and dominant ions. The application of factor analysis gave four factors, namely the salinization factor, anthropogenic/secondary enrichment factor, the secondary and the micro-nutrient fertilizers, and the aluminum fertilizer factor. These factors give insights about the possible sources of mineralization in the studied water. The hydrogeochemical study of the groundwater revealed that the hydrogeochemical processes controlling the groundwater chemistry in the study area are mainly affected by the groundwater occurrence either to the east (the old cultivated areas) or to the west (the new reclaimed area) of Bahr Youssef Canal. In the new reclaimed lands, the hydrogeochemical processes affecting the groundwater are the silicate weathering, and the ion exchange process with the aquifer materials, in addition to the infiltration of irrigation water that is enriched in Ca^2+^ and SO_4_^2−^ resulting in the Ca-SO_4_ water type dominating these areas. Conversely, in the old reclaimed lands, the mixed Ca–Mg–Cl and the Na-Cl water types characterize the groundwater in this area where the dominant reactions are ion exchange, silicate weathering, gypsum applications in soil, and evaporation.

The groundwater quality evaluation indicates that the water quality varies from fair to excellent for the drinking and domestic purposes, where the best water is located in the northern and central parts of the study area. The suitability of groundwater for irrigation was evaluated using several indices indicating that the groundwater is suitable for irrigation in the northwest and western parts of the study area. However, in the north, central, and northeastern parts, care should be considered because of increased KR, Na%, and unsuitable US Salinity classes, especially in samples G2, G13, G15, G16, and G18. As some groundwater samples lie in high salinity classes on the US Salinity diagram, it is recommended to use this water for plants with good salt tolerance under good drainage conditions. Based on CR for transport of groundwater through pipes, all groundwater samples are corrosive except samples G10, G13, G15, and G17. So, it is favored to use corrosion resistance pipes for transporting these waters.

The use of statistical analysis provided data reduction and classification that is subsequently used as a base for hydrogeochemical analysis.

## Supplementary Information

Below is the link to the electronic supplementary material.

Supplementary file1 (DOCX 40 KB)

## Data Availability

The data used in generating this work are available from the authors upon request.
