# Supplementary material for: Unraveling the hydrogeochemical evolution and pollution sources of shallow aquifer using multivariate statistical analysis and hydrogeochemical techniques: a case study of the Quaternary aquifer in Beni Suef area, Egypt
Source: Environ Monit Assess. 2023 May 15;195(6):670. doi: 10.1007/s10661-023-11206-9 (PMC10185637; doi:10.1007/s10661-023-11206-9)
Supplement: Supplementary file 1 — Supplementary file1 (DOCX 40 KB) [file 10661_2023_11206_MOESM1_ESM.docx]

**Table S1. Results of the major and trace element analysis of the collected water samples (Element concentrations and TDS are in mg/l, EC is in µS/cm, temperature is in degree Celsius)**

| Water Type | Hole ID | Temp. | pH | EC | CO_3-_ | HCO_3_^-^ | Total Alkalinity | TDS | Ca^2+^ | K^+^ | Mg^2+^ | Na^+^ | Cl^+^ | PO_4_^3-^ | SO_4_^2+^ |
| --- | --- | --- | --- | --- | --- | --- | --- | --- | --- | --- | --- | --- | --- | --- | --- |
| Ca-Mg-Cl type | G1 | 22.6 | 7.39 | 3410 | 0 | 278 | 278 | 2295 | 330 | 34 | 59.4 | 290 | 855.87 | < 0.2 | 287.07 |
|  | G11 | 24.5 | 7.04 | 1457 | 0 | 351 | 351 | 948 | 101.07 | 16 | 29.16 | 160 | 149 | < 0.2 | 259.94 |
|  | G12 | 24.4 | 7.32 | 1322 | 0 | 354 | 354 | 846 | 89.13 | 12 | 36.43 | 148 | 189.62 | < 0.2 | 119.2 |
|  | G14 | 23 | 7.23 | 1400 | 0 | 360 | 360 | 904 | 96.88 | 13 | 34.99 | 152 | 199.22 | < 0.2 | 125.49 |
|  | G17 | 22.4 | 7.33 | 1310 | 0 | 410 | 410 | 842 | 80.73 | 10 | 38.88 | 140 | 190.62 | < 0.2 | 118.39 |
| Na-Cl type | G2 | 25.4 | 6.88 | 7770 | 0 | 385 | 385 | 5056 | 586 | 32 | 48 | 1067 | 1488 | 11.37 | 1252 |
|  | G15 | 21.8 | 7.42 | 1869 | 0 | 660 | 660 | 1208 | 131.04 | 19 | 33.53 | 250 | 197.16 | < 0.2 | 278.41 |
|  | G16 | 24.2 | 7.31 | 1796 | 0 | 400 | 400 | 1154 | 134.04 | 18 | 34.11 | 245 | 310.26 | < 0.2 | 186.87 |
|  | G18 | 22.6 | 7.24 | 2710 | 0 | 385 | 385 | 1752 | 154.9 | 22 | 43.13 | 355 | 465 | < 0.2 | 325 |
| Ca-Mg-SO_4_ type | G3 | 24.1 | 7.04 | 3640 | 0 | 361 | 361 | 2400 | 492.9 | 17 | 24.18 | 295 | 341 | 3.15 | 1056 |
|  | G4 | 24.3 | 7.08 | 3540 | 0 | 326 | 326 | 2304 | 472.7 | 12 | 13.6 | 310 | 466.85 | 2.67 | 1044.11 |
|  | G5 | 24.8 | 7.4 | 2330 | 0 | 200 | 200 | 1550 | 432.8 | 11 | 20.55 | 105 | 147.02 | 4.04 | 989.1 |
|  | G6 | 23.6 | 7.24 | 3220 | 0 | 302 | 302 | 2112 | 480.8 | 10 | 19.25 | 250 | 221.81 | < 0.2 | 1113.09 |
|  | G7 | 23.8 | 6.95 | 5140 | 0 | 326 | 326 | 3424 | 669 | 14 | 19 | 489 | 571.08 | < 0.2 | 1545 |
|  | G8 | 27.2 | 7 | 3660 | 0 | 263 | 263 | 2406 | 448 | 11 | 25 | 294 | 346.37 | < 0.2 | 1021 |
|  | G9 | 28.4 | 6.92 | 2520 | 0 | 175 | 175 | 1642 | 402 | 10 | 14 | 155 | 149.86 | < 0.2 | 957 |
| Ca-HCO_3_ type | G10 | 23.4 | 7.14 | 608 | 0 | 302 | 302 | 388 | 42.18 | 8 | 21.08 | 52 | 21.55 | < 0.2 | 52.86 |
| Na-Ca-HCO_3_ type | G13 | 23.2 | 7.22 | 932 | 0 | 360 | 360 | 600 | 55.01 | 8 | 18 | 148 | 88 | < 0.2 | 78.21 |

**S1 Continued**

| Water Type | Hole ID | | | Al | | Ba | | Cu | | Fe | | Mn | | Zn | | Cr | Co | | Ni | | V | |
| --- | --- | --- | --- | --- | --- | --- | --- | --- | --- | --- | --- | --- | --- | --- | --- | --- | --- | --- | --- | --- | --- | --- |
| Ca-Mg-Cl type | G1 | | | 0.098 | | 0.18 | | 0.039 | | 0.788 | | 0.387 | | 0.033 | | < 0.002 | 0.005 | | 0.024 | | < 0.001 | |
|  | G11 | | | 0.136 | | 0.107 | | 0.079 | | 0.099 | | 0.024 | | 0.019 | | < 0.002 | 0.004 | | < 0.004 | | 0.006 | |
|  | G12 | | | 0.042 | | 0.093 | | 0.044 | | 0.215 | | 0.129 | | 0.008 | | < 0.002 | 0.006 | | 0.006 | | 0.015 | |
|  | G14 | | | 0.053 | | 0.085 | | 0.089 | | 0.153 | | 0.437 | | 0.022 | | < 0.002 | < 0.003 | | 0.004 | | < 0.001 | |
|  | G17 | | | 0.304 | | 0.085 | | 0.058 | | 0.267 | | 0.433 | | 0.026 | | < 0.002 | 0.003 | | 0.01 | | < 0.001 | |
| Na-Cl type | G2 | | | 0.156 | | 0.025 | | 0.13 | | 0.088 | | 0.099 | | 0.058 | | 0.013 | < 0.003 | | 0.007 | | < 0.001 | |
|  | G15 | | | 0.019 | | 0.179 | | 0.045 | | 0.062 | | 1.14 | | 0.013 | | < 0.002 | 0.003 | | < 0.004 | | < 0.001 | |
|  | G16 | | | 0.179 | | 0.16 | | 0.047 | | 0.231 | | 0.468 | | 0.005 | | < 0.002 | 0.006 | | 0.006 | | < 0.001 | |
|  | G18 | | | 0.088 | | 0.149 | | 0.044 | | 0.113 | | 0.644 | | 0.026 | | < 0.002 | < 0.003 | | < 0.004 | | 0.006 | |
| Ca-Mg-SO_4_ type | G3 | | | 0.053 | | 0.026 | | 0.065 | | 0.144 | | 0.334 | | 0.422 | | < 0.002 | 0.006 | | 0.013 | | < 0.001 | |
|  | G4 | | | 0.14 | | 0.039 | | 0.176 | | 0.178 | | 0.168 | | 0.062 | | < 0.002 | 0.003 | | 0.008 | | < 0.001 | |
|  | G5 | | | 0.151 | | 0.074 | | 0.064 | | 0.467 | | 0.333 | | 0.02 | | < 0.002 | 0.003 | | 0.008 | | < 0.001 | |
|  | G6 | | | 0.059 | | 0.041 | | 0.048 | | 0.263 | | 0.298 | | 0.02 | | < 0.002 | 0.002 | | 0.015 | | < 0.001 | |
|  | G7 | | | 0.069 | | 0.029 | | 0.085 | | 0.086 | | 0.449 | | 0.042 | | < 0.002 | 0.003 | | 0.008 | | < 0.001 | |
|  | G8 | | | 0.085 | | 0.032 | | 0.14 | | 0.132 | | 0.084 | | 0.046 | | 0.003 | < 0.003 | | 0.008 | | < 0.001 | |
|  | G9 | | | 0.173 | | 0.033 | | 0.208 | | 0.19 | | 0.127 | | 0.076 | | 0.002 | < 0.003 | | 0.006 | | < 0.001 | |
| Ca-HCO_3_ type | G10 | | | 0.084 | | 0.086 | | 0.059 | | 0.137 | | 0.011 | | 0.015 | | < 0.002 | 0.003 | | < 0.004 | | < 0.001 | |
| Na-Ca-HCO_3_ type | | G13 | 0.081 | | 0.079 | | 0.075 | | 0.147 | | 0.506 | | 0.021 | | < 0.002 | | 0.003 | < 0.004 | | < 0.001 | |  |

**Table S2. WQI_CCME_ rank categories and color code representation (modified after CCME, (2001))**

| **Meaning** | **Percent** | **Grade** | **Color code** |
| --- | --- | --- | --- |
| Water quality is protected with virtual absence of threat or impairment; conditions very close to natural or pristine levels | **94 - 100 %** | **Excellent** | **Green** |
| Water quality is protected with only minor degree of threat or importance; conditions rarely depart from natural or desirable levels | **79 - 94 %** | **Good** | **Blue** |
| Water quality is usually protected but occasionally threatened or impaired; conditions sometimes depart from natural or desirable levels | **64 - 79 %** | **Fair** | **Yellow** |
| Water quality is frequently threatened or impaired; conditions often depart from natural or desirable levels | **44 -64 %** | **Marginal** | **Brown** |
| Water quality is almost always threatened or impaired; conditions usually depart from natural or desirable levels | **0 - 44 %** | **Poor** | **Red** |

**Table S3. Classification of water quality for irrigation purposes based on several parameters**

| **Parameter** | **Range** | **Water Quality** | **Reference** |
| --- | --- | --- | --- |
| **KR** | equal or less than 1 | Good | (Kelly, 1940) |
|  | more than 1 | Unsuitable |  |
| **Na %** | < 20 | Excellent | (Wilcox, 1948) |
|  | 20-40 | Good |  |
|  | 40-60 | Permissible |  |
|  | > 60 | Undesirable |  |
| **SAR** | < 10 | Excellent | (Wilcox, 1948) |
|  | 10-18 | Good |  |
|  | 18-26 | Fair |  |
|  | > 26 | Unsuitable |  |
| **RSC** | < 1.25 | Safe | (Eaton, 1950; Wilcox et al., 1954) |
|  | 1.25-2.5 | Marginal |  |
|  | > 2.5 | Unsuitable |  |
| **MR** | > 50 | Unsuitable | (Paliwal, 1972) |
|  | < 50 | Suitable |  |
| **CR** | < 1 | Safe | (Raman, 1985) |
|  | > 1 | Unsafe |  |

KR (Kelly’s ratio) RSC (Residual Sodium Carbonate)

Na % (Sodium percent) MR (Magnesium ratio)

SAR (Sodium absorption ratio) CR (Corrosivity ratio)

**Table S4. Classes of USSL diagram (Zaman et al.*,* 2018)**

| **Salinity Hazard** | |
| --- | --- |
| **C1** | Used for irrigation of most crops on most soils with little development of soil salinity. |
| **C2** | Used if a moderate infiltration can occur. Plants with moderate salt tolerance can be grown without special salinity control. |
| **C3** | Cannot be used on soil with restricted drainage and poor infiltration, special salinity control may be required and plants with good tolerance should be selected. |
| **C4** | Not suitable for irrigation under ordinary conditions but may be used in special cases (soil must be permeable, drainage must be good and irrigated water must be applied in excess to provide considerable infiltration). Only very salt tolerance plant should be selected. |
| **Sodium Hazard** | |
| **S1** | Used for irrigation under ordinary circumstances on all soil with little development of harmful levels of sodium content, whereas sensitive sodium crops as avocados and stone fruit trees may accumulate harmful concentrations of sodium. |
| **S2** | It shows a remarkable sodium hazard in fine textured soils which have cation exchange capacity and low infiltration unless gypsum is applied in the soil. This water may be used in coarse textured soil or organic soils with good permeability. |
| **S3** | It produces harmful sodium content in most soils. Its use requires good drainage, high infiltration, high organic conditions, and soil amendments. Chemical amendments may be not suitable for very high salinity waters. |
| **S4** | It is unsatisfactory for irrigation uses except at low and perhaps medium salinity. Gypsum as soil amendment may facilitate the use of this class in irrigation. |

**Table S5. The suitability of groundwater for irrigation**

| Hole ID | KR | SAR | Na% | RSC | Mn (mg/l) | USSL | Suitability |
| --- | --- | --- | --- | --- | --- | --- | --- |
| G10 | 0.59 | 1.63 | 39.19 | 1.12 | 0.01 | C2S1 | Suitable |
| G11 | 0.94 | 3.61 | 49.82 | -1.66 | 0.02 | C3S1 |  |
| G12 | 0.87 | 3.34 | 47.60 | -1.62 | 0.13 | C3S1 |  |
| G14 | 0.86 | 3.37 | 47.45 | -1.79 | 0.44 | C3S1 |  |
| G17 | 0.85 | 3.21 | 46.82 | -0.48 | 0.43 | C3S1 |  |
| G1 | 0.59 | 3.86 | 38.77 | -16.73 | 0.39 | C4S2 | Suitable with Caution |
| G3 | 0.48 | 3.52 | 33.35 | -20.59 | 0.33 | C4S2 |  |
| G4 | 0.55 | 3.84 | 35.88 | -19.29 | 0.17 | C4S2 |  |
| G5 | 0.20 | 1.34 | 17.27 | -19.94 | 0.33 | C4S1 |  |
| G6 | 0.43 | 3.04 | 30.38 | -20.55 | 0.30 | C4S1 |  |
| G7 | 0.61 | 5.09 | 38.29 | -29.50 | 0.45 | C4S2 |  |
| G8 | 0.53 | 3.66 | 34.93 | -20.03 | 0.08 | C4S2 |  |
| G9 | 0.32 | 2.07 | 24.86 | -18.28 | 0.13 | C4S1 |  |
| G2 | 1.40 | 11.41 | 58.79 | -26.78 | 0.10 | C4S4 | Unsuitable |
| G13 | 1.53 | 4.43 | 61.18 | 1.69 | 0.51 | C3S1 |  |
| G15 | 1.17 | 5.05 | 55.06 | 1.55 | 1.14 | C3S2 |  |
| G16 | 1.13 | 4.90 | 54.00 | -2.91 | 0.47 | C3S2 |  |
| G18 | 1.37 | 6.51 | 58.73 | -6.60 | 0.64 | C4S2 |  |

**Table S6. Mean concentrations of the studied parameters of the distinguished clusters (Element concentrations and TDS are in mg/l, EC is in µS/cm)**

|  | **C1** | **C2** | **C3** |
| --- | --- | --- | --- |
| **pH** | 7.26 | 7.09 | **7.84** |
| **EC** | 1681 | **3436** | 442 |
| **HCO_3_^-^** | **386** | 279 | 167 |
| **TDS** | 1094 | **2263** | 283 |
| **Ca^2+^** | 121 | **485** | 45.53 |
| **K^+^** | **16** | 12.14 | 4.38 |
| **Mg^2+^** | **34.87** | 19.37 | 12.41 |
| **Na^+^** | 194 | **271** | 24.03 |
| **Cl^-^** | 267 | **321** | 30.04 |
| **PO_4_^3-^** | 0.1 | **1.5** | 0.1 |
| **SO_4_^2-^** | 183 | **1104** | 32.34 |
| **Al** | 0.108 | 0.104 | **2.13** |
| **Ba** | 0.120 | 0.039 | **0.139** |
| **Co** | 0.004 | 0.003 | **0.046** |
| **Cu** | 0.058 | 0.112 | **0.116** |
| **Fe** | 0.221 | 0.209 | **0.817** |
| **Mn** | **0.418** | 0.256 | 0.124 |
| **Zn** | 0.019 | 0.098 | **0.525** |
